# Hahnemann and Cholera Microbes

**Published:** 1884-12

**Authors:** R. E. Dudgeon

**Affiliations:** London


### HOMŒOPATHIC PHYSICIAN.

A MONTHLY JOURNAL OF MEDICAL SCIENCE.

"If our school ever gives up the strict inductive method of Hahnemann, we are lost, and deserve only to be mentioned as a caricature in the history of medicine."—CONSTANTINE HERING.

Vol. IV. DECEMBER, 1884. No. 12.

### HAHNEMANN AND CHOLERA MICROBES.

R. E. DUDGEON, M. D., LONDON.

Everything Dr. P. P. Wells writes can hardly fail to be interesting and instructive, and this is the character of the article he contributes to your October number on *Cholera, Snake-bite, and their Lessons*, but surely he for once has made a mistake in saying (p. 272), when contrasting the testimony of Hahnemann respecting cholera with that of modern "scientific medicine," "And yet, let it be remembered, with no thought of 'microbes' he found the cure."

Dr. Wells must have quite forgotten—or perhaps he has never read—the pamphlet published by Hahnemann in 1831, entitled *Appeal to Thinking Philanthropists Respecting the Mode and Propagation of the Asiatic Cholera*, which I have translated in the *Lesser Writings of Hahnemann.* In that remarkable work he says (p. 851 of *L. W.*) that the cause of cholera is undoubtedly an invisible cloud "composed of probably millions of these miasmatic animated beings, which at first developed on the broad, marshy banks of the tepid Ganges," etc. ; again : "The cholera-miasm * * grows into an enormously increased brood of those extraordinarily minute, invisible, living creatures, so inimical to human life, of which the contagious matter of the cholera most probably consists."

These passages show that Hahnemann was a thorough believer in the cholera "microbe," though he had never seen it, microscopic research not having been so far advanced in 1831 as it is to-day.

But not only was Hahnemann a believer in an organized germ being the cause of cholera; his recommendation of Camphor was partly, at least, founded on his belief that it was the true germicide of the cholera microbe. At page 854 (*L. W.*) he says, talking of camphorated spirit: "This medicine, which alone is efficacious, and which most certainly destroys the miasm about the patient;" and again, "By the cure of the disease with pure Camphor, they would at the same time eradicate and annihilate the miasm (that probably consists of innumerable, invisible living beings)."

The dose, too, in which he directs the Camphor to be given shows that he trusted chiefly to its germicidal power. Here are his directions, taken from another essay by him *On the Cure and Preventing the Asiatic Cholera* (*Lesser Writings*, p. 846): "The patient must get as often as possible (at least every five minutes), a drop of spirits of Camphor (made with one ounce of Camphor to twelve of Alcohol) on a lump of sugar or in a spoonful of water. Some spirit of Camphor must be taken in the hollow of the hand and rubbed into the skin of the arms, legs, and chest of the patient; he may also get a clyster of half a pint of warm water mixed with two full teaspoonfuls of spirit of Camphor, and from time to time some Camphor may be allowed to evaporate on a hot iron."

The pathogenesis of Camphor certainly shows a great similarity to the symptoms of the first stage of cholera, but it is evident Hahnemann was not guided to its use solely by this similarity, or he would hardly have prescribed it in such comparatively enormous doses, unless he had considered such doses as necessary for the destruction of the cholera microbes. Indeed, he hints at the beginning of this last-named essay that he was led to give Camphor on account of its recorded success in the treatment of Asiatic cholera at Dünaburg.

So Hahnemann must be looked on as a firm believer in, if not the actual inventor of, the microbe theory of cholera, and the original proposer of its treatment by means of a germicide remedy. Whether the hypothesis is true I cannot, of course, decide, but, true or not, it was held by Hahnemann, as he distinctly says, and his Camphor treatment of cholera is founded on his belief in its truth.

